# Tribbles in the 21st Century: The Evolving Roles of Tribbles Pseudokinases in Biology and Disease

**DOI:** 10.1016/j.tcb.2016.11.002

**Published:** 2017-04

**Authors:** Patrick A. Eyers, Karen Keeshan, Natarajan Kannan

**Affiliations:** 1 Department of Biochemistry, Institute of Integrative Biology, University of Liverpool, Liverpool, L69 7ZB, UK; 2 Paul O’Gorman Leukemia Research Centre, Institute of Cancer Sciences, College of Medical, Veterinary and Life Sciences, University of Glasgow, Glasgow, G12 0YN, UK; 3 Institute of Bioinformatics, University of Georgia, Athens, GA 30602, USA; 4 Department of Biochemistry and Molecular Biology, University of Georgia, Athens, GA 30602, USA

**Keywords:** Tribbles, Trb, TRIB, TRIB1, TRIB2, TRIB3, pseudokinase, signaling, cancer, evolution, ubiquitin, E3 ligase

## Abstract

The Tribbles (TRIB) pseudokinases control multiple aspects of eukaryotic cell biology and evolved unique features distinguishing them from all other protein kinases. The atypical pseudokinase domain retains a regulated binding platform for substrates, which are ubiquitinated by context-specific E3 ligases. This plastic configuration has also been exploited as a scaffold to support the modulation of canonical MAPK and AKT modules. In this review, we discuss the evolution of TRIBs and their roles in vertebrate cell biology. TRIB2 is the most ancestral member of the family, whereas the emergence of TRIB3 homologs in mammals supports additional biological roles, many of which are currently being dissected. Given their pleiotropic role in diseases, the unusual TRIB pseudokinase conformation provides a highly attractive opportunity for drug design.

## Trends

Pseudoenzymes are inactive counterparts of classical enzymes and have evolved in all kingdoms of life, where they regulate a vast array of biological processes. The pseudokinases are one of the best-studied families of human pseudoenzymes.

Eukaryotic TRIB pseudokinases evolved from a common ancestor (the human TRIB2 homolog), and contain a highly atypical pseudokinase domain fused to a unique docking site in an extended C tail that binds to ubiquitin E3 ligases.

TRIB evolution has led to the appearance of three mammalian TRIB pseudokinases, termed TRIB1, TRIB2, and TRIB3, which contain both unique and shared features.

In cells, TRIB pseudokinases act as modulators of substrate ubiquitination and as molecular scaffolds for the assembly and regulation of signaling modules, including the C/EBPα transcription factor and AKT and ERK networks.

TRIB1 and TRIB2 have potent oncogenic activities in vertebrate cells, and recent evidence also suggests that TRIB2 acts as a tumour suppressor, consistent with the requirement for balanced TRIB signaling in the regulation of transcription, differentiation, proliferation, and apoptosis.

## Introduction and Historical Perspective

**Figure 1:**
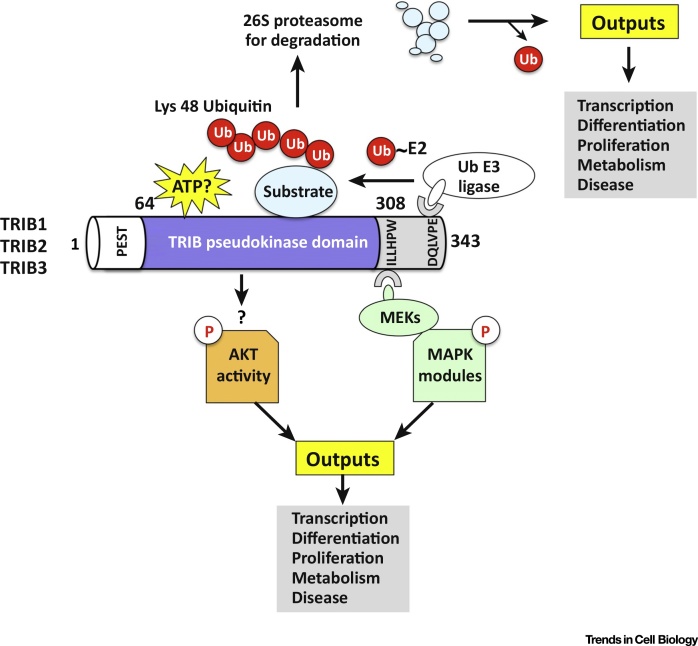
Classical Tribbles (TRIB) Signaling Features. All TRIB polypeptides contain a variable N-terminal ‘PEST’ domain, a pseudokinase domain lacking a canonical ‘DFG’ (metal-binding) motif, and a unique C-tail, which contains two key regulatory elements. The HPW[F/L] motif at the beginning of the pseudokinase C-tail targets MAPKK/MEK family members, giving TRIBs the potential to regulate and/or integrate distinct stress and proliferative MAPK modules. The conserved structural C-terminal DQXVP[D/E] peptide motif supports a direct association with E3 ubiquitin ligases, including COP1, which specifies K48-linked ubiquitin chains in substrates such as C/EBPα, thereby regulating transcription factor stability via the ubiquitin proteasome system. TRIBs can also modulate AKT/FOXO signaling modules, although the molecular details for individual pseudokinases remain to be clarified. Overall, a series of shared mechanisms contribute to TRIB-regulated signaling pathways that support cell context-specific programs of cellular differentiation, proliferation, and survival. Abbreviations: P, phosphate; Ub, ubiquitin.

Protein kinases and phosphorylation modulate all aspects of eukaryotic cell biology and, together with members of the Ubiquitin system, have become highly significant for mechanistic drug targeting [1]. Post-translational modification of proteins permits regulatory flexibility and endows them with the ability to control signaling networks through combinatorial mechanisms. Consistently, crosstalk between phosphorylation and ubiquitination is thought to be widespread in eukaryotic signaling [2]. The three TRIB pseudokinases (TRIB1, TRIB2, and TRIB3) represent a prominent subbranch of eukaryotic pseudoenzymes that are unique within the human kinome 3, 4. TRIB proteins regulate intracellular cell signaling and appear to have evolved two major mechanisms of action. The first exploits conserved adaptions in the ancient eukaryotic protein kinase (ePK) fold to position protein ‘substrates’ and control their E3 ligase-dependent ubiquitination, likely through a switch-like pseudokinase mechanism. The second involves a more obscure scaffolding function, which operates to integrate and modulate signals flowing into and through canonical MAPK and AKT modules (Figure 1). Accordingly, TRIBs are fundamental regulators of cell cycle, differentiation, metabolism, proliferation, and cell stress, as reflected by the appearance of several excellent reviews in these specific areas 5, 6, 7, 8, 9.

TRIB pseudokinases represent a subbranch of the CAMK subfamily in the human kinome [3] and, although a shared eukaryotic evolutionary origin is apparent from bioinformatic comparisons 10, 11, 12, the molecular basis for their specific evolutionary trajectory and associated cellular functions has not been evaluated in depth. Indeed, several critical questions in the TRIB field remain (see Outstanding Questions). For example, it is important to uncover clues as to why three distinct TRIB pseudokinase polypeptides evolved in human cells and how they mechanistically support diverse regulatory and disease-associated signaling pathways (Figure 1). An analysis of these issues represents a central focus of this review.

## Origin and Evolution of Tribbles Pseudokinases

In mammalian cells, the three related TRIB family members (TRIB1–3) are classed as serine/threonine pseudokinases that either lack (TRIB1), or have low, (TRIB2 and TRIB3) vestigial ATP affinity and phosphotransferase capacity when analyzed *in vitro* 10, 13, 14. In many cells, TRIB1 and 2 pseudokinases are rather unstable cellular proteins, likely due to conserved destabilizing motifs present in the small N-terminal PEST region 15, 16. The adjacent TRIB pseudokinase domain is linked to a short C-terminal ubiquitin E3 ligase-targeting motif, which was recently proposed to interact *in cis* with the TRIB regulatory pseudokinase domain 14, 17. In addition, the pseudokinase domain has evolved structurally [18] to correctly position and regulate potential substrates for ubiquitylation by a variety of classes of Ubiquitin E3 ligases [19]. The formation of regulated multiprotein complexes that then dictate cellular signaling is a recurring theme in TRIB biology (Figure 1). However, while the roles of TRIB pseudokinases in cellular signaling, metabolism, and disease are increasingly appreciated, little is known about their early origins and the specific signaling requirements that have shaped them during evolutionary history.

TRIB proteins derive their name from the single metazoan fly gene ‘Tribbles’ (*Trbl*), which encodes a pseudokinase with developmental roles in this model genetic organism. *Trbl* was discovered through three independent *Drosophila* screens, which revealed genes and mutations that affect either oogenesis via Slbo, the alpha ortholog of the mammalian CCAAT enhancer-binding protein (C/EBPα) transcription factor [20] or gastrulation during embryogenesis via String, the fly ortholog of the CDC25 phosphatases 21, 22. Of major interest, Trbl mutant cells exhibit premature mitosis, leading to defective gastrulation based on an inability to degrade target proteins in a timely fashion. Trbl fly models continue to be instrumental in revealing requirements for the pseudokinase domain in biological signaling events that have been conserved in vertebrate eukaryotes 23, 24. For example, String is the *Drosophila* ortholog of the CDC25 dual-specificity phosphatases, which initiate eukaryotic S-phase and mitosis and are themselves regulated at various cell cycle checkpoints. Recent data confirmed that TRIB2 protein expression is also cell cycle regulated in human cells, positioning it as a potential modulator of CDC25 phosphatases, which it degrades through a ubiquitin- and proteasome-dependent mechanism [25]. Fly Trbl was also reported as an interacting partner for the proto-oncogene AKT, and this interaction also appears to be conserved among the metazoa, where TRIB3 binding directly modulates mTORC2-dependent AKT activation 26, 27, 28, 29, seemingly without affecting AKT stability. TRIB2 has also been described as a dosage-dependent suppressor of the AKT-phosphosubstrate FOXO, specifically as a modulator of the cytoplasmic localization of FOXO3a in human cells [30]. Interestingly, a TRIB3-regulated AKT-FOXO transcriptional network also operates in human neurons [27], where TRIB3 expression levels are inversely correlated with the Parkinson's disease-associated ubiquitin E3 ligase PARKIN [31]. Indeed, TRIB3, but not TRIB1 or TRIB2, mRNA expression is upregulated in the developing murine brain [32] and TRIB3 immunostaining is markedly increased in sections of substantia nigra from Parkinson's disease brains [31]. The relatively mild cognitive and memory brain phenotypes observed in a C57BL/6 TRIB3-deficient mouse model [31] support the notion that normalizing pathological increases in TRIB3 levels in neurodegenerative disease might be an interesting new medical strategy.

**Figure 2:**
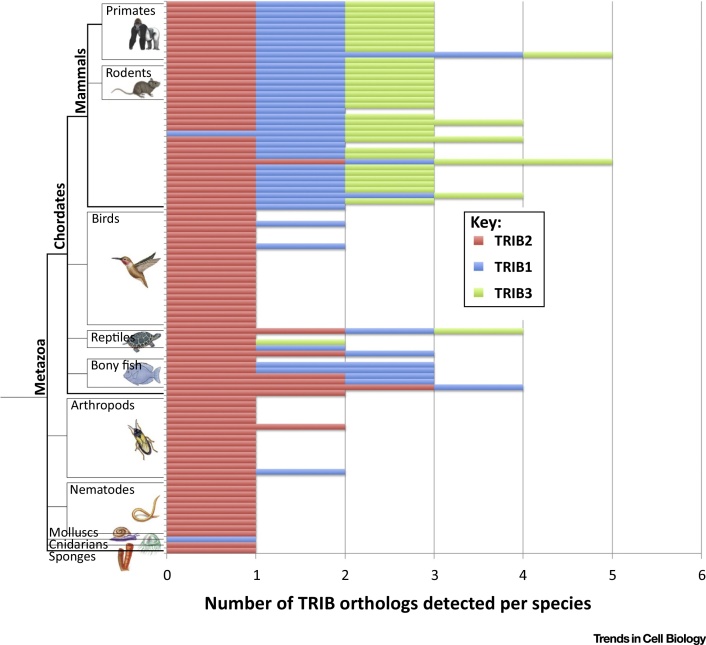
Taxonomic Coverage and Analysis of Tribbles (TRIB) Family Kinases. The number of TRIB1, TRIB2, and TRIB3 orthologs detected across all the major metazoan species for which accurate genomic kinome data are available are shown. Each row represents a single species and the number of TRIB orthologs identified in each species is indicated. Orthologs were identified by scanning a hierarchical sequence profile of diverse ePKs and TRIB family members against sequenced proteomes contained in the latest nonredundant proteome sequence set in Uniprot (downloaded October 2016) using the MAPGAPS program [93]. The most significant hits to TRIB1, TRIB2, and TRIB3 profiles were annotated as putative orthologs. Fragmentary sequences of less than 150 amino acids in length were filtered out. Reptilian TRIB3 orthologs were detected in larger sequence databases (NCBI nr, EST) and all three TRIB pseudokinases are observed in reptilian species (including alligator) in the corresponding Uniprot proteome sequence set.

To address major knowledge gaps in how the functional specialization of TRIB genes occurred during evolution, we identified and classified TRIB-related sequences from all the major taxonomic groups where they are present (Figure 2). Taxonomic analysis of these sequences demonstrates that they are almost entirely confined to the animal kingdom, and are absent in non-metazoan eukaryote kinomes, including those annotated in fungi, plants, and choanoflagellates. We speculate that this represents a shift towards ubiquitination-based substrate regulation by the TRIB pseudokinase domains in metazoan eukaryotes, although other explanations are possible. As shown in Figure 2, the statistically derived TRIB2 signature sequence is the earliest (most ancestral) member among the TRIB1, 2, and 3 family. Consistently, orthologs of TRIB2 (but not TRIB1 or TRIB3) can readily be detected in the oldest metazoans, such as cnidarians (*Nematostella vectensis*) and sponges (*Amphimedon queenslandica*), whereas TRIB1 and TRIB3 orthologs are restricted to specific later metazoan lineages that led to the vertebrates, where all three TRIBs pseudokinases are consistently preserved. It is likely that the emergence of TRIB1 and TRIB3 at later stages of evolution was driven by unique regulatory requirements associated with signaling integration by these proteins in higher organisms. TRIB1 likely appeared through TRIB2 gene duplication during the diversification of vertebrates from invertebrates, since multiple copies of TRIB2 and a single copy of TRIB1 are observed in non-mammalian chordates, such as fish (Actinopterygii) and reptiles. Remarkably, the TRIB3-specific sequence signature appears in all mammalian lineages, and from the extensive data analyzed here, emerged relatively recently during the diversification of mammals from non-mammalian chordates (Figure 2). While most mammalian species harbor at least one copy of TRIB1, 2, and 3, some encode multiple copies, suggesting functionally relevant gene duplications; an extreme version being the marmoset *Callithrix jacchus*, whose genome encodes three copies of TRIB1, one copy of TRIB2, and 2 copies of TRIB3. The cellular consequences of these duplications are obscure, although we speculate that they represent a requirement for new biological functions associated with higher vertebrates, perhaps including unique TRIB3 roles in highly evolved brains, which might be driven by increased gene dosages of this specific pseudokinase. Whatever their specific functions, TRIB genes have likely been coopted into evolutionarily appropriate biological roles that depend on their unique pseudokinase properties compared with catalytically active kinases (Box 1), which have been defined previously 3, 10, 13, 33, 34, 35, 36.

## What Defines A Tribbles Pseudokinase?

**Figure 3:**
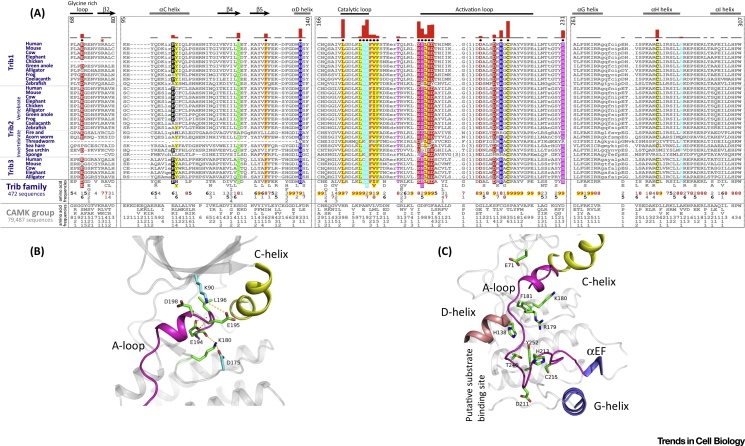
Distinguishing Sequence and Structural Features of Tribbles (TRIB) Pseudokinases. (A) Constraints that help distinguish TRIB kinases from all other kinases are shown in a contrast hierarchical alignment, where representative TRIB kinases from diverse organisms constitute the display alignment; all TRIB-like sequences available (492 sequences, September 2016) constitute the foreground alignment, and related CAMK sequences (79 487 sequences) constitute the background alignment. Complete foreground and background alignments are not shown due to the hundreds of text pages required. Instead, information encoded in these large alignments is shown as residue frequencies directly below the display alignment where, for example, the number ‘5′ indicates that the corresponding residue occurs 50–60% of the time at the corresponding position. The histogram above the alignment plots the strength of the selective pressure shifting residues at each position in the TRIB kinases away from the residue composition observed at the corresponding positions in CAMKs. Residue positions subject to the strongest constraints are highlighted with chemically similar amino acids colored similarly; weakly conserved positions and nonconserved positions are shown in dark and light gray, respectively. Dots below the histograms indicate those residue positions that most strikingly distinguish TRIB kinases from CAMKs as selected by the Bayesian pattern partitioning procedure [94]. Key secondary structural elements are indicated above the alignment; amino acid numbering corresponds to human TRIB2. Identifiers for TRIB sequences used in the display alignment can be compared with canonical kinase sequences by inspecting Box 1. (B,C) Modeling of the structural disposition of TRIB-specific residues forming the regulatory activation loop, atypical DFG motif (E[S/N]LED), and αC helix in human TRIB2. The kinked αC-helix is shown in yellow and the activation loop (A-loop) is shown in magenta. TRIB family conserved residues are shown in green. Putative hydrogen-bonding interactions in the modeled structure are shown by broken lines and the putative substrate-binding site in the C-lobe is labeled. Structural image was generated using PyMoL. Specific TRIB gene identifiers are listed in the legend to Figure 4.

**Figure 4:**
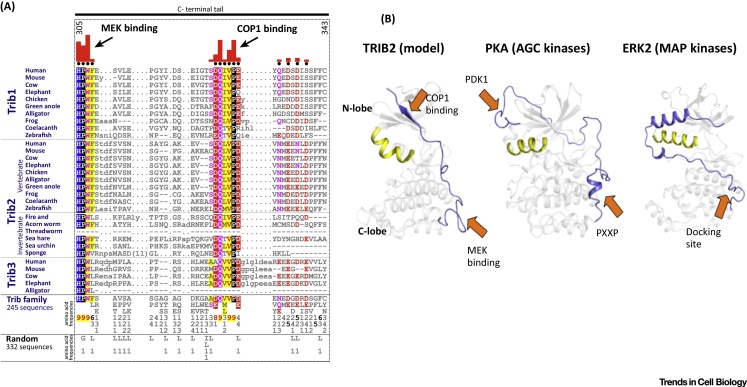
The C-tail, A Unique ‘Degrading’ Feature of Tribbles (TRIB) Pseudokinase Polypeptides. (A) Alignment of TRIB pseudokinase C-tail segment highlighting key conserved residues and motifs found in eukaryotic TRIB pseudokinases. See Figure 2 legends for details**.** Unique TRIB gene identifiers are: Trib1 Human Q96RU8; Trib1 Mouse Q8K4K4; Trib1 Cow A6QLF4; Trib1 Elephant G3T9X9; Trib1 Chicken H9L0P6; Trib1 Green anole G1KJZ8; Trib1 Alligator A0A151P7Z3; Trib1 Frog F7BWB1; Trib1 Coelacanth H3ALB4; Trib1 Zebrafish E7FD70; Trib2 Human Q92519; Trib2 Mouse Q8K4K3; Trib2 Cow Q5GLH2; Trib2 Elephant G3TC04; Trib2 Chicken Q7ZZY2; Trib2 Green anole G1K8G5; Trib2 Alligator A0A151N8V7; Trib2 Frog Q76D08; Trib2 Coelacanth H3A37; Trib2 Zebrafish E7F3S2; Trib2 Fire ant XP_011171156.1; Trib2 Acorn Worm XP_002742313.1; Trib2 Threadworm A0A0K0E067; Trib2 Sea hare XP_005101496.1; Trib2 Sea urchin XP_792075.2; Trib2 Sponge I1G1T0; Trib3 Human Q96RU7; Trib3 Mouse Q8K4K2; Trib3 Cow Q0VCE3; Trib3 Elephant G3SZ76; Trib3 Alligator A0A151NVV1. (B) Common mechanism for structural tethering of the C-tail to the kinase domain in TRIB2 (model based on TRIB1 X-ray analysis, Protein Data Ban ID: 5CEM) and two canonical kinase families, with PKA representing the AGC kinases [39] and ERK2 representing the MAP kinases [95]. All three subfamilies of (pseudo)kinase are regulated by conformational changes in the C-terminal tail that directly engage the kinase domain *in ci*s.

TRIB pseudokinases are predicted to be three-domain proteins containing an N-terminal PEST region, a pseudokinase domain (containing an unusual N-lobe and a canonical C-lobe) and a C-terminal COP1-binding peptide region, which interacts *in cis* with a pocket formed adjacent to the unusual αC-helix found in the TRIB pseudokinase domain (Figure 1). Recent structural analysis of the human TRIB1 pseudokinase domain [14] confirms an atypical kinase fold that diverges most notably in the N-lobe, which harbors most of the catalytic machinery, but which preserves a putative substrate-binding site in the C-lobe of the kinase domain. No structural information has been reported for the N-terminal PEST domain, or for any domains of the TRIB2 or TRIB3 pseudokinases. Although these same defining regions are conserved across TRIB polypeptides when comparing the pseudokinase domain with catalytically active CAMK relatives (Box 1), the availability of tens of thousands of protein kinase-like sequences from diverse organisms provides a timely opportunity to define distinguishing or unique features of TRIB pseudokinases using statistical comparisons of large data sets. Such approaches across kinomes have previously been invaluable to provide new insights into protein kinase structure, function, and evolution 37, 38, 39, 40, 41, 42. As collated in Figure 3, a Bayesian statistical comparison reveals strong selective constraints imposed on each of the individual TRIB kinase sequences during evolution. At a gross level, these constraints correspond to ‘TRIB-specific’ residues and/or motifs that are highly conserved in TRIB kinases but strikingly different in the closely related pseudokinase SgK495 and the broader family of canonical CAMKs. We focus on the most distinctive features in the core (conserved) regions of the TRIB family, namely the pseudokinase domain (Figure 3A) and flanking C-terminal tail (Figure 4A). In passing, we note that the N-terminal PEST domain contains distinct sequence motifs that are characteristic of each TRIB variant, and these subfamily specific motifs are assumed to regulate distinct protein turnover patterns in both ‘normal’ and disease-associated cells 43, 44.

Within the pseudokinase domain, TRIB-specific constraints are imposed on residues and/or motifs that are dispersed in primary sequence, but that spatially cluster in two critical regions of the pseudokinase domain (Figure 3A), namely the active site and C-terminal substrate-binding lobe. The divergent nature of active site residues in TRIB pseudokinases has been noted in previous studies and in the recently solved crystal structure of TRIB1 [14], where these residues mediate specific hydrogen bonding and van der Waals interactions (Figure 3B) that stabilize the activation loop in a unique inactive conformation that serves to preclude nucleotide binding in the TRIB1 ATP-binding site [14]. In contrast to the pseudoactive site, TRIB-specific divergence in the C-lobe has not been evaluated in depth, despite the overall structural similarity of TRIB1 to the C-lobe of canonical CAMKs. Some of the TRIB-specific residues of unknown functions in the C-lobe include the D[KR]H[GA]C motif in the activation loop, H138 in the D-helix, and a conserved cysteine residue in the αH helix (Figure 3B,C). In the crystal structure of TRIB1, the region corresponding to the D[KR]H[GA]C motif in the activation segment is disordered. However, modeling of the activation loop in both an active and inactive conformation suggests that TRIB-specific residues in the C-lobe potentially stabilize the activation loop in an autoinhibitory conformation that might occlude substrate binding, as previously revealed by structural analysis of TRIB1 [14]. The C-lobe is a universal docking site for protein substrates in canonical kinases and autoinhibitory conformations involving the activation loop have been observed in multiple kinases, including canonical CAMKs [45]. Thus, it is likely that a near-universal regulatory kinase mechanism is also at play in the TRIB pseudokinases, and that TRIB-specific constraints in the pseudokinase domain reflect specific variations on a common mechanistic autoregulatory theme found in most, if not all, kinases.

## A Unique C-Terminal Regulatory Region in TRIB Pseudokinases

In addition to characteristic residues and/or motifs in the pseudokinase domain, the critical C-terminal tail involved in protein–protein interactions is readily identified as a distinguishing feature of TRIB kinases; this segment is highly conserved in TRIB kinases, but strikingly different in kinases outside of the TRIB subfamily (Figure 4A). No detectable similarity is observed at this locus between TRIB primary sequences and any other evolutionarily related protein kinases, defining the tail as a statistically unique feature. The TRIB C-terminal tail is defined by two unique sequences: the HPW[F/L] and DQXVP[D/E] motifs near the extreme N and C-terminal regions of the C-tail, respectively (Figure 4A). The HPW[F/L] motif is involved in binding of MEK1 (and other MAPKK dual-specificity kinases) to TRIB pseudokinases 46, 47, 48, whereas the DQXVP[D/E] motif is intimately involved in COP1 binding in all TRIB proteins 49, 50 and required to drive tumorigenesis in leukemia models [51]. Although these motifs are not observed in the C-terminal tail of other protein kinases, they are predicted to engage with the TRIB pseudokinase domains in a manner analogous to canonical, well-studied protein kinases, such as PKA and MAP kinases, which utilize these flanking regions to drive regulatory mechanisms involved in kinase activation and substrate phosphorylation (Figure 4B). This common regulatory theme, evident between a divergent pseudokinase family (modeled as TRIB2) and multiple canonical kinase families in which allosteric coupling is established (represented by AGC kinases and MAP kinases), reinforces how flanking sequences outside the (pseudo)kinase domain appear to have been repeatedly used to permit switch-like regulation of the kinase fold. Of particular interest for the TRIBs, this mechanism is predicted to be independent of catalysis, although the phosphorylation of a highly specific localized cellular substrate is challenging to rule out completely.

Interestingly, the HPW[FL] motif is tethered to the C-lobe of the pseudokinase domain in the recently solved TRIB1 crystal structure, while the DQXVP[D/E] motif is tethered to the N-lobe. Why would such tethering of the C-tail to the pseudokinase domain be important for TRIB functions? One possibility is that this interaction allows efficient coupling of COP1 binding (or other regulatory binding proteins) in the C-tail with coopted substrate-binding functions associated with the degenerate pseudokinase domain. Such a view may also explain why the C-helix and activation loop conformation in the TRIB1 (and presumably TRIB2 and TRIB3) pseudokinase domain are stabilized in a unique conformation [14], since potential conformational changes in these regions can be allosterically coupled to the COP1-binding site, which is absolutely required for substrate ubiquitination. Indeed, the binding of a TRIB1 C-tail-derived peptide sequence to a COP1-motif peptide is of a measurably higher affinity when compared side-by-side with a TRIB1 protein containing the C-tail [18], the latter retaining coupled pseudokinase and C-tail interactions. Thus, protein allostery, rather than phosphorylation-based catalysis more commonly associated with canonical protein kinases, might be the key driving force for TRIB kinase evolution and functional specialization. In turn, this suggests that TRIBs evolved to be pseudokinases that exploit a noncanonical (but still bilobal) protein kinase fold that modulates cellular ubiquitination through protein–protein interactions. The reuse of a pseudokinase domain to mediate signaling has also been noted in other evolutionarily distinct pseudoenzymes, including the PAN3 and ADCK3 pseudokinases, which have refined the kinase fold to scaffold enzyme-catalyzed processes as diverse as mRNA deadenylation [52] and Coenzyme Q biosynthesis [53]. In support of a coevolutionary hypothesis in TRIB pseudokinases, it is interesting to note that some TRIB2 orthologs (such as a pathogenic thread worm) lack detectable sequence similarity in the C-tail sequence, and also diverge in complementary C-tail docking regions in the pseudokinase N-lobe (modeled as deletions in alignment Figure 3, Figure 4). Since parasitic threadworms kinomes are currently poorly annotated, we cannot rule out that these sequences either exist or have become cryptic at the amino acid level. Nonetheless, we suspect that characterizing unusual TRIB variants and potential mechanisms of action in a variety of model organisms will be important for fully understanding TRIB functions in both normal human biology and disease.

## Tribbles Links to Cancer: A Corruption of Cell Signaling?

In humans, the TRIB gene family has been implicated in many different cancers, but especially in melanoma, lung, liver, and acute leukemias 5, 6. The molecular basis of these disease links is still in the process of being dissected in a variety of cell types and model systems. However, a major mechanistic function of TRIBs in cancer cells appears to be the (inappropriate) association of TRIB proteins with substrate degradation and stability networks, leading to a subsequent imbalance in timely regulation of crucial transcriptional networks. For example, TRIB2-mediated degradation of the transcription factor C/EBPα is known to have an oncogenic role in the development of acute myeloid leukemia (AML) 54, 55, lung [56], and liver cancers 57, 58. These studies all point to abnormal regulation of TRIB transcription, translation, or protein turnover as disease drivers, and below we use the hematological system and other cancer models to describe how this is thought to work mechanistically at the cellular level in mammals.

### TRIB Pseudokinase Function in Myeloid and Lymphoid Systems

**Figure 5:**
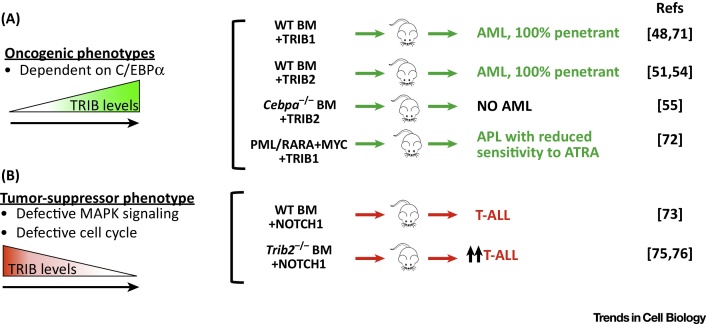
Tribbles 1 (TRIB1) and TRIB2 Pro- and Anti-tumorigenic Activities Associated with Vertebrate Leukemias. (A) Murine cancer models in which TRIB1 and TRIB2 function as oncogenes when TRIB1 or TRIB2 are overexpressed (+TRIB1,  + TRIB2) leading to fully penetrant, fatal acute myeloid (AML, green), but not lymphoid (T cell acute lymphoblastic; T-ALL) leukemias. Elevated TRIB1 expression also contributes to chemotherapy resistance (a protumorigenic response) in APL (indicated in a PML/RARA + MYC model). (B) Mouse models in which the loss of TRIB2 reveals a tumor-suppressive activity. This is shown through homozygous TRIB2 knockout, which results in an accelerated lymphoid (T-ALL induced by active NOTCH1, red), but not myeloid, leukemia. Abbreviations: ATRA, *all*-*trans*-retinoic-acid, an APL therapy; APL, acute promyelocytic leukemia (a subtype of AML); BM, bone marrow; Cebpa^–/–^, C/EBPα-knockout mouse; Trib2^–/–^, Trib2-knockout mouse; WT, wildtype mouse. Citations refer to *in vivo* mouse models of leukemia demonstrating oncogenic or tumor-suppressive TRIB biology.

The *Trib2* gene was first identified as a murine myeloid oncogene, since its overexpression in a bone marrow transplant model leads to the development of a potent transplantable AML with 100% penetrance and short latency [54]. In this model, TRIB2 preferentially degrades the p42 isoform of the myeloid transcription factor C/EBPα, which leaves the truncated oncogenic p30 isoform (which lacks the canonical TRIB-binding site identified in the TRIB1 crystal structure) intact [54]. TRIB1 (but not TRIB3) functionally resembles TRIB2 in this phenotypic mouse cancer model, since it also degrades C/EBPα and causes highly penetrant AML (Figure 5), in line with a postulated TRIB evolutionary pathway that led linearly between TRIB2 and TRIB1 and, hence, on to TRIB3 (Figure 2) and the high level (71%) of amino acid similarity between TRIB1 and TRIB2 within the pseudokinase domain. The functional difference between TRIB pseudokinases in these systems is further highlighted by their differential expression in hemopoietic cells [59], with TRIB2 highest in the lymphoid cell compartment, TRIB1 highest in the myeloid cell compartment, and TRIB3 expression constant across all cell types examined. Interestingly, the human *TRIB1* gene is located at the same chromosomal locus (8q24.13) as the *MYC* oncogene and, therefore, *MYC* (and potentially *TRIB1)* cancer susceptibility genes are co-amplified in a significant percentage of human tumours, where the 8q24 amplicon is the most commonly amplified region across multiple cancer types [60]. Furthermore, an ‘oncogenic’ R107L TRIB1 mutation in the pseudokinase domain has been identified in a Down syndrome-related AML [7]; this Arg residue is broadly conserved across the TRIB pseudokinases (Figure 3A, asterisk), but is a Leu residue in the canonical kinase PKA, where it packs up against hydrophobic residues in the C-terminal tail [39]. Interestingly, when this amino acid was analyzed across the kinome [61], an Arg or Lys residue was found in approximately 35% of human kinases, with Leu representing the most common single amino acid, accounting for approximately 25% of (pseudo)kinases in the human ePK family. Although mechanistic effects of an R107L substitution are not fully understood in TRIB1, enhancement of both ERK phosphorylation and C/EBPα degradation have been demonstrated in an R107L TRIB1 murine bone marrow model of AML [62]. Based on the crystal structures of PKA and TRIB1, we speculate that this mutation contributes to abnormal TRIB1 pseudokinase function by disrupting regulatory protein interactions with interacting partners, such as MAPKK family members [46], Ubiquitin E3 ligases, or *cis*-acting flanking regulatory segments in TRIB itself. Of related interest, *TRIB1* gene polymorphisms are also associated with nonalcoholic liver and metabolic syndromes [63], consistent with a relatively specialized function for TRIB1 in lipid metabolism 8, 43.

In the hematopoietic system, C/EBPα is essential for granulopoiesis [64] and TRIB1 and TRIB2-mediated degradation of C/EBPα p42 blocks this differentiation process. Mechanistically, degradation of C/EBPα p42 by TRIB2 is known to occur via a proteasome-dependent pathway involving lysine 48 polyubiquitination [55]. Using mouse genetics, it was shown that the presence of C/EBPα is paradoxically required for TRIB2-induced AML, and only in the presence of the C/EBPα p42 isoform is a cooperative effect observed with TRIB2 and C/EBPα p30 [55]. Structure–function analysis of TRIB2 revealed that deletion or mutation of the TRIB signature C-terminal E3 ligase COP1-binding site (Figure 4A) prevented TRIB2-mediated degradation of C/EBPα and this correlated directly with a failure to induce AML *in vivo* [51]. Interestingly, TRIB1, 2, and 3 all retain COP1-binding motifs in their C-tails (Figure 4A), but TRIB3 alone fails to drive C/EBPα degradation. This finding is consistent with a unique (or only partially overlapping) set of target substrates for TRIB3 in comparison to TRIB1 and TRIB2. However, preserved functionality in the C-tail in TRIB3 is confirmed by its ability to facilitate cellular COP1-mediated ubiquitination and degradation of Acetyl CoA carboxylase, the rate-limiting enzyme in fatty acid synthesis [50]. Distinct ubiquitin E3 ligases have also been associated with TRIB2 signaling, including TRIM21 in a lung cancer model [56]. In liver cancer models, TRIB2 overexpression conversely stabilizes the co-activator YAP, an oncogenic transcription factor, via binding to the distinct E3 ubiquitin ligase β-TRCP 57, 58. Intriguingly, and in contrast to COP1 and TRIM21, TRIB2 interaction with β-TRCP leads to the inhibition instead of promotion of substrate degradation; it remains unclear whether post-translational modifications control the targeted interaction of different TRIB pseudokinases with specific ubiquitin ligases. However, in terms of feedback regulation, TRIB2 stability has also been shown to be regulated at the protein level in liver cancer cells, in part due to the downregulation of the E3 ligase SMURF1 [16].

Through their interaction with ubiquitylated transcription factors, or regulation of signaling pathways that culminate in cell type-specific reprogramming, TRIB pseudokinases are regulated by, and fundamental regulators of, gene transcription networks. An analysis of TRIB1, 2, and 3 promoters confirms that TRIB1 and 2 both have putative E2F and C/EBPα transcription factor-binding sites among a large number of conserved canonical transcription factor sequence determinants (Box 2). Consistently, TRIB2 has been shown to be part of a regulatory loop involving E2F1 and TRIB2, in which E2F1 binds to the *TRIB2* gene promoter to drive expression of the TRIB2 protein, which is then positively reinforced via p30 C/EBPα expression of E2F1 [65]. In T cell leukemia models, the Paired homeobox transcription factor PITX1 was shown to regulate TRIB2 [66]. In addition, aberrant TAL-1 activation, which is detected in up to 60% of T cell acute lymphoblastic leukemias (T-ALLs) [67], was shown to transcriptionally target TRIB2 [68], allowing TRIB2 to positively reinforce an oncogenic transcriptional program involving GATA3, RUNX1, MYB, and E2A, potentially balancing the oncogenic and tumor-suppressive biological activities of these factors [69].

Despite being capable of driving myeloid leukemia when overexpressed alone, it is assumed that cooperating lesions occur in TRIB1 and TRIB2 mouse leukemia models (Figure 5). Putative cooperating genes in TRIB1 and TRIB2-mediated AML include HOX pathway genes, such as *HOXA9*, *MEIS1*, *NUP98*-*HOXD13*, and *C/EBPAp30* 48, 55, 70, 71. Gene expression analysis has revealed that *TRIB2* expression levels, while generally low in AML, are higher in *PML-RARA*-positive leukemia than in *PML-RARA*-negative leukemia [59]. As described, the *TRIB1* gene is located on chromosome 8, the most common chromosomal gain in human AML and APL (distinguished by the fusion oncogene PML/RARA). The hypothesis that TRIB1 and PML/RARA cooperate has been tested and, while they do not cooperate to drive a shorter latency leukemia *in vivo*, TRIB1 and PML/RARA have functionally redundant inhibitory effects on C/EBPα, which also impacts responses [72] to therapeutic All-*Trans* Retinoic Acid (ATRA, Figure 5).

The association of TRIB2 with a subset of AMLs with dysregulated C/EBPα is especially intriguing, since T lymphoid genes, including *NOTCH1* [73] and *CD7*, as well as *CD34*, a stem cell gene, also associate with this subset [74]. It is interesting to speculate that TRIBs may have a role in lineage decisions or phenotypic characteristics of myeloid and lymphoid leukemias, which may be determined by their specific cellular substrates. While it was shown that TRIB2 is a transcriptional target of the T cell transcription factor NOTCH1 [74], the absence of TRIB2 in a murine TRIB2-knockout model [75] was found to accelerate NOTCH1-driven T-ALL (Figure 5). This work also revealed a novel tumor suppressor-like function for TRIB2 in the cell cycle and proliferation of early T cell progenitor cells, and associated high levels of TRIB2 expression with early immature T-ALL and deregulated MAPK signaling [75]. TRIB2 is cyclically expressed during the cell cycle and has the ability to promote the proteasomal degradation of the mitotic regulator CDC25C, which might explain some of the uncontrolled proliferation characteristics of leukemia [25]. It was subsequently confirmed in an independent study that this tumor-suppressor activity of TRIB2 is lymphoid cell specific [76].

## TRIB Pseudokinase Functions in other Model Cancer Systems

Further evidence for TRIB2 as a modulator of tumorigenic activity comes from liver cancer cells, where the overexpression of TRIB2 was shown to negatively regulate WNT signaling activity, leading to inhibition of cell growth [19]. The cell models in this study have wildtype (WT) β-catenin, and the authors confirmed that TRIB2 overexpression reduced WNT-mediated transcriptional activity and decreased levels of β-catenin and TCF4 protein. Consistent with this model, the loss of TRIB2-impaired liver cancer cell survival *in vitro* and *in vivo* [57] is associated with a dominant β-catenin mutation. Interestingly, the broad analysis of all cancers failed to identify conserved mutations or chromosomal alterations involving TRIB2. By contrast, its elevated expression is strongly associated with cancer prognostics [77]. Indeed, *TRIB2* has been identified as a candidate biomarker for melanoma diagnosis and progression, because it exhibits low expression in healthy skin samples, increases in benign melanoma, with the highest expression seen in malignant melanoma samples [78]. In addition, TRIB1 expression has been shown to be essential for the survival of prostate cancer cells and is linked with an aggressive disease phenotype and poor prognosis [79]. TRIB1 is also involved in the etiology of glioma [80], breast [81], ovarian [82], and follicular thyroid cancer [83]. TRIB3 expression was found elevated in patients with colorectal cancer and its expression correlated with poor overall survival [84]. *TRIB3* gene expression, in contrast to TRIB3 protein expression [85], has been shown to correlate with poor prognosis in patients with breast cancer [86] and a poor prognosis in patients with non-small cell lung cancer (NSCLC) [87].

## Concluding Remarks

The appearance and retention of pseudoenzymes during evolution in essentially all enzyme families suggests that these domains are malleable templates that can be coopted for new biological functions when required 88, 89, 90. This is clearly demonstrated within the human protein kinase superfamily by the three TRIB pseudokinases, which together control large networks of cellular signaling pathways, many of which are known to be dysregulated in disease. In this review, we have highlighted the importance of classifying and analyzing each TRIB family member as a unique pseudokinase variant, and this is most clearly shown by comparing their evolutionary and disease-associated biology in vertebrates. The evolution of TRIB1 and TRIB3 from a common TRIB2 ancestor is particularly interesting and, by using unique structural and mechanistic features, TRIB pseudokinases appear to have evolved a set of fundamental biological roles, some of which are shared, at least in the simplistic experimental cell and animal models evaluated thus far. In the future, proteomic and cell biology approaches will permit the accurate dissection of the common and specific TRIB machinery that brings about the three TRIB pseudokinase signaling modules. The mechanisms that underlie the ability of TRIB proteins to function as oncogenes or tumor suppressors are currently not well understood. However, these are likely to be linked to their complex functions in cell proliferation, protein degradation, transcriptional regulation, and canonical signaling pathway modulation and might also be cell context dependent, impacting the cellular fate of both normal and tumor cells. Indeed, these signaling pathways, and the TRIB pseudokinases and protein–protein interactions that regulate them, present new and potentially important pharmacological opportunities for therapeutic intervention 91, 92 in both metabolic and proliferative disorders.

Outstanding Questions What is the structural basis for the distinct cellular roles of TRIB pseudokinases? In particular, how do subtle variations in sequence identified in the three distinct, but related, pseudokinase domains drive cell signaling? Will a combination of X-ray and NMR approaches be needed to evaluate TRIB dynamics in the complete polypeptide in comparison to the isolated pseudokinase domain?

What are the specific binding partners of the three TRIB pseudokinase polypeptides, within and across the PEST region, the pseudokinase domain and the C-tail? Can these regions be trapped with substrates bound and studied by mass spectrometry, and are all substrates that bind actively ubiquitinated? Furthermore, is this process controlled on the pseudokinase, or in a processive manner after release? Finally, how many different ubiquitin E3 ligases do the TRIB pseudokinases engage?

What is the role (if any) of the vestigial ATP binding detected in human TRIB2 and TRIB3 *in vitro*? This is an important question, since the concentration of ATP in cells is in the mM range, which might be sufficient for TRIB pseudokinases to have nucleotide-dependent mechanisms, as appears to be the case for several other pseudokinases. In this context, do TRIB pseudokinases undergo switching mechanisms that change the accessibility of the highly atypical nucleotide-binding site, coupling it to ubiquitination?

Leading on from this, is the atypical nucleotide-binding site suitable for targeting with small molecules? Do known protein kinase inhibitors have unappreciated ‘off-target’ effects on TRIB pseudokinases in cells? Can small molecules be designed to probe TRIB structural dynamics and cell biology? In particular, can compounds be identified that promote changes in TRIB stability in different cell types? These classes of chemical might be useful leads for new types of drug.

How are the expression levels of TRIB pseudokinases regulated under physiological conditions, and before and during pathology? Related to this, what are the transcriptional networks in operation that fulfill the obligations of the TRIB proteins in eukaryotes, and how have these changed during evolution?

## Figures and Tables

**Figure I:**
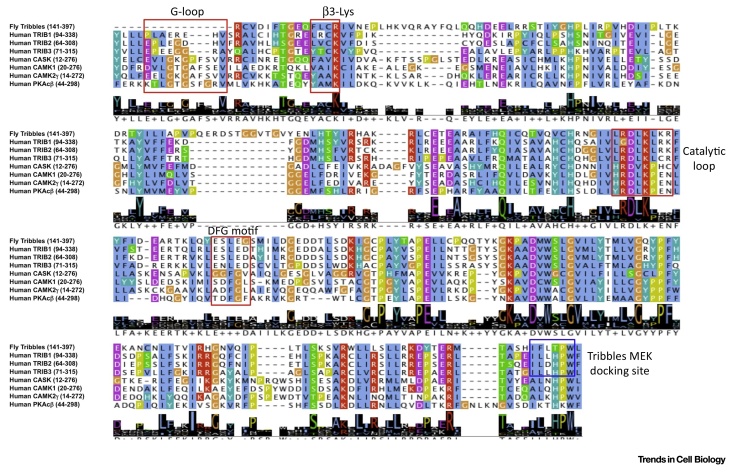
CAMK Subfamily Sequence Alignment of selected (Pseudo)Kinase Domains.

**Figure I:**
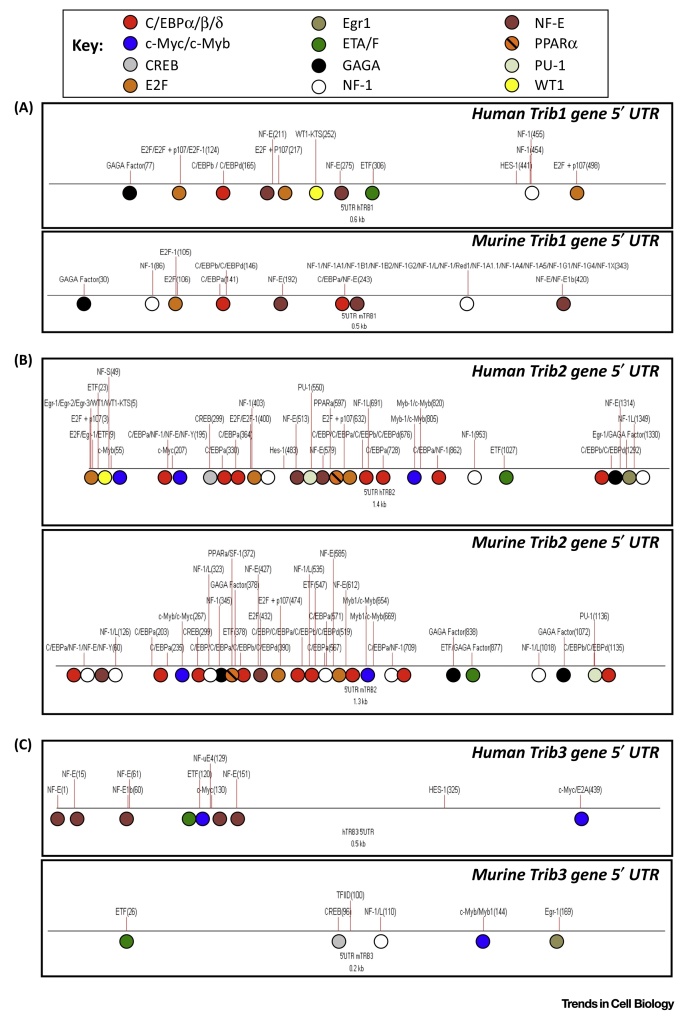
Transcription Factor-Binding Sites in vertebrate *TRIB* Gene Promoters.

## Box 1 CAMK Subfamily Sequence Alignment within the (Pseudo)Kinase Domain

Figure I shows an Alignment and Sequence Logo amino acid conservation plot of *Drosophila* TRIB (Trbl), human TRIB1–3, human CASK (a CAMK-related pseudokinase), canonical human CAMK1α, CAMK2γ, and the benchmark PKAcβ AGC kinase domains. A comparison of sequences around the degraded TRIB glycine-rich loop, the conserved β3 lysine, the unusual TRIB catalytic loop, and the metal-binding ‘DFG’ motif (including the TRIB-specific E[S/N]LED sequence) are boxed in red. Conserved amino acid motifs at these positions together define the TRIB pseudokinase domain signature (applied in Figure 2, Figure 3, main text). The HPW[F/L] MEK binding site at the junction of the TRIB pseudokinase C-lobe and C-tail is boxed in blue. Numbering refers to amino acid boundaries within each human (pseudo)kinase domain.

## Box 2 Predicted Transcription Factor-Binding Sites in Human and Murine TRIB Gene Promoter Regions

Transcription factor-binding sites were predicted using Transcription Element Search System (TESS) software [96], based on the sequence of the extended 5′ untranslated regions (UTR) sequenced from human and murine TRIB1 (Figure IA), TRIB2 (Figure IB), or TRIB3 (Figure IC) genetic loci. Twelve canonical transcription factor-binding sites identified in the two sets of promoters are colored according to the key. Note that several functional E2F1 and C/EBPα-binding sites on the TRIB2 promoter predicted in this analysis have been experimentally verified [65]. The differences in length of the 5′UTR region of each promoter analyzed are indicated for reference. Note the relatively large predicted number of transcription factor-binding sites for TRIB2 compared with the relatively small number of predicted transcription factor binding sites for TRIB3.
